# Ferroptosis-Related Gene Contributes to Immunity, Stemness and Predicts Prognosis in Glioblastoma Multiforme

**DOI:** 10.3389/fneur.2022.829926

**Published:** 2022-03-10

**Authors:** Jiawei Dong, Hongtao Zhao, Fang Wang, Jiaqi Jin, Hang Ji, Xiuwei Yan, Nan Wang, Jiheng Zhang, Shaoshan Hu

**Affiliations:** ^1^Department of Neurosurgery, The Second Affiliated Hospital of Harbin Medical University, Harbin, China; ^2^Cancer Center, Department of Neurosurgery, Zhejiang Provincial People's Hospital, Affiliated People's Hospital, Hangzhou Medical College, Hangzhou, China

**Keywords:** ferroptosis, glioblastoma multiforme, immunity, stemness, prognosis

## Abstract

Ferroptosis, a recently discovered regulated programmed cell death, is associated with tumorigenesis and progression in glioblastoma. Based on widely recognized ferroptosis-related genes (FRGs), the regulation of ferroptosis patterns and corresponding characteristics of immune infiltration of 516 GBM samples with GSE13041, TCGA-GBM, and CGGA-325 were comprehensively analyzed. Here, we revealed the expression, mutations, and CNV of FRGs in GBM. We identified three distinct regulation patterns of ferroptosis and found the hub genes of immunity and stemness among DEGs in three patterns. A prognostic model was constructed based on five FRGs and verified at the mRNA and protein level. The risk score can not only predict the prognosis but also the degree of immune infiltration and ICB responsiveness by functional annotation. The overall assessment of FRGs in GBM patients will guide the direction of improved research and develop new prognostic prediction tools.

## Introduction

Grade IV glioma, which is termed as glioblastoma multiforme (GBM), is the most lethal glioma (1). Despite advances in the treatment of GBM with surgery, radiation, and chemotherapy, the survival rate of patients remains 18 months (2). Previous investigations have depicted some malignant biological features that contribute to the highly recurrent and drug-resistance of GBM (3). Tremendous research studies have focused on molecular markers that contribute to GBM stemness and immunity (4, 5). Our previous study conducted a comprehensive analysis of the stemness of GBM (6). However, many therapies targeting these molecular markers become less effective in clinical practice. Therefore, the novel and effective prognostic models for the prediction of GBM prognosis and immunotherapy response need to be investigated and clarified.

Ferroptosis is a new type of programmed cell death proposed by Stockwell et al. (7). Research on the significance of ferroptosis in cancer has recently gained momentum, whereas disruption of this process under human intervention may show clinical effects (8, 9). Ferroptosis manifests cell membrane rupture and blebbing, mitochondrial and morphological changes, with the cell nucleus remain intact (10). For instance, downregulation of SLC1A5 provides melanoma cell partial immunity to ferroptosis induction (11). GOT1 inhibition promotes pancreatic cancer cell death by potentiating the activity of ferroptosis (12). Knockdown of TFRC can inhibit the cell proliferation of BRCA cell lines (13). More and more genes related to ferroptosis have been identified in glioma, such as ACSL4 that protects glioma cells and exerts antiproliferative effects by activating a ferroptosis pathway (14), ATF3 that contributes to brucine-induced glioma cell ferroptosis (15), and COPZ1 that manipulates NCOA4 to regulate the ferroptosis process in GBM (16). These ferroptosis-related genes (FRGs) are closely linked to tumorigenesis and progression. However, whether these genes are associated with the prognostic value and molecular functions of GBM patients has not been elucidated.

Despite its implication in cell death, recent studies also evaluated ferroptosis-associated diseases and their role on immunity. For example, CD8^+^ T cells suppress tumor growth by inducing ferroptosis and pyroptosis (17). In addition, ferroptosis could release various damage-associated molecular patterns (DAMPs) or lipid metabolites that are involved in the cellular immune response (18). Notably, ferroptosis was associated with tumor immune checkpoints in clear cell renal cell carcinoma (19). These researches explored the mechanisms in ferroptosis and immune microenvironment. However, these studies have not specifically focused on GBM, and the relationship between ferroptosis and immune response in GBM has not been well characterized.

Herein, we integrated data from the public Gene Expression Omnibus (GEO), The Cancer Genome Atlas (TCGA) databases, and the Chinese Glioma Genome Atlas (CGGA) to evaluate the role of FRGs signature in the prognosis in GBM patients. We further identified three distinct regulations of ferroptosis. Comparison of the DEG of three patterns unveiled five key genes involved in immunity and stemness. These genes may have potential value in the regulation of ferroptosis in GBM. Finally, a risk score based on FRGs had been constructed. Within functional annotation, we found that the risk score is not only a good predictive value for survival but also a potential factor for immune checkpoint blockade (ICB) responsiveness. Our study could help to guide the link between ferroptosis and GBM stem cells intensive research in the future and identify new ferroptosis-related targets and immune therapies.

## Materials and Methods

### Data Acquisition

Raw RNA-seq data (FPKM files) and clinical data on GBM were extracted from The Cancer Genome Atlas (TCGA, https://portal.gdc.cancer.gov/), Chinese Glioma Genome Atlas (CGGA, http://www.cgga.org.cn/) and Gene Expression Omnibus (GEO, https://www.ncbi.nlm.nih.gov/geo). After data filtration, GSE13041, TCGA-GBM, and CGGA-325 with 516 GBM tissue samples were gathered in this study for further analysis. Infiltration estimation for all TCGA tumors was collected from TIMER2.0 (http://timer.comp-genomics.org/). Copy number variant (CNV) data and somatic mutations of all genes were downloaded from the UCSC Xena browser (https://xenabrowser.net). CNV differences of all genes were calculated by the chi-square test (*p* < 0.05). The location of the significantly different genes on the chromosomes was shown by the RCircos R package. The protein–protein interaction network was produced by the STRING (https://www.string-db.org) database and was reconstructed *via* Cytoscape software. The protein expressions in human normal tissues and tumor tissues were validated *via* the Human Protein Altas (HPA, https://www.proteinatlas.org/).

### Identification of FRGs

Ferroptosis-related genes had been categorized according to the existing literature which contains iron metabolism, oxidant metabolism, lipid metabolism, energy metabolism, and other unclassified factors (9, 20–22). According to the description of FRGs in glioma research (23), 59 genes were incorporated into follow-up studies and were provided in Supplementary Table 1. Considering the small number of normal brain tissues in the TCGA, we investigated the expression of the FRGs on the online web server GEPIA (http://gepia.cancer-pku.cn/). Differentially expressed genes (DEGs) were calculated using the R package “LIMMA” (|logFC| > 1 and *p* < 0.05).

### Functional Enrichment Analyses

To functionally annotate DEG sets during the analysis, Kyoto Encyclopedia of Genes and Genomes (KEGG), pathway analysis, and Gene Ontology (GO) were performed in R software version 4.0.3 using ClusterProfiler package. To calculate mRNA expression-based stemness index (mRNAsi), we used the OCLR algorithm constructed by Malta's team (24). The mRNAsi was represented using an index between zero to one to signal that the higher the mRNAsi, the greater activity of cancer stem cells. The CytoHubba plugin version 0.1 in Cytoscape version 3.8.2 was employed to identify hub genes, and enrichment analysis was performed using the ClueGO plugin version 2.5.8.

The TIMER, CIBERSORT, QUANTISEQ, Microenvironment Cell Populations-counter (MCP-counter), XCELL, and Estimating the Proportion of Immune and Cancer cells (EPIC) algorithms were used to estimate the abundance of immune cells between the high- and low-risk groups. The “ESTIMATE” R package was used to assess immune infiltration (based on the ImmuneScore, StromalScore, and ESTIMATEScore). The clustering was performed using WGCNA and the module–trait correlations with mRNAsi, EREG-mRNAsi, and ESTIMATEScore. According to the number of the genes, the minModuleSize of the mRNA was set to 50. Gene sets that could predict the responses to immune checkpoint blockade therapy were obtained from the work by Mariathasan. Single-sample gene set enrichment analysis (ssGSEA) was used to estimate immune-related functions in TCGA-GBM patients utilizing gene set variation analysis (GSVA) (25) version 1.40.1.

Tumor immune dysfunction and exclusion (TIDE) (http://tide.dfci.harvard.edu/), a well-established algorithm was employed to predict the clinical response to ICB therapy (26). TIDE is a computational framework developed to evaluate the potential of tumor immune escape from the gene expression profiles of cancer samples. The TIDE score could serve as a surrogate biomarker to predict response to ICB. The SubMap (https://www.genepattern.org/) was employed to validate the reliability of the prediction of TIDE. Mapping result is represented as a subclass association matrix filled with *p*-values for each subclass association (27).

### Construction of a Scoring System and Calculate the Risk Score

Univariate Cox regression analysis was implemented to filtrate the prognostic FRGs. The ConsensusClusterPlus package in R was employed to investigate the detailed information in unsupervised subclasses discovery and to divide samples into appropriate parts for maximum stability (28). Thereafter, we used the R package “glmnet” to conduct least absolute shrinkage and selection operator (LASSO) Cox regression algorithm and development of a potential risk signature. The minimum value of lambda was derived from 1,000 crossvalidations (“1-se” lambda), which corresponding partial likelihood deviance value was the smallest for the risk model (29, 30). At last, coefficients with regression were confirmed by the “cvfit” function with 1,000 repeats. FRG prognostic signature involves five genes. The risk score calculating formula is as follows:


$$\begin{array}{l} Riskscore=\overset{n}{\underset{i=\;1}{\sum }}Coef_{i}\;*\;x_{i} \end{array}$$

where Coe*f*_*i*_ means the coefficients and *x*_*i*_ is the expression value of each FRGs. This formula was used to calculate the risk score for each GBM patient. The predictive ability of prognostic signature for clinical traits and survival was reflected by receiver operating characteristic (ROC) and the area under the curve (AUC).

The independent clinical factor validated by univariate and multivariate Cox regression analyses was enrolled to construct a nomogram for prognosis prediction. Patients with missing data were excluded. The nomogram was performed using the “survival” and “regplot” packages of R 4.1.0 to investigate the probability of 1-, 3-, and 5-year overall survival (OS).

### RNA Extraction and Real-Time PCR

For RNA extraction, three GBM tissues and one peritumoral brain edema were collected in the Second Affiliated Hospital of Harbin Medical University. This research was approved by all the patients and the ethics committee of hospital. Total RNA was isolated using TRIzol reagent (Invitrogen, USA) according to the manufacturer's instructions. According to the manufacturer's instructions of the Nanodrop ND-2000 spectrophotometer (Thermo Scientific, USA), 2 μg of the total RNA was transcribed into cDNA. SYBR Green PCR kit (Takara, Japan) was used for qRT-PCR. The 2–ΔΔCq method was used to calculate gene transcription level, with β-actin mRNA as control. Data represent the mean ± SD of triplicate real-time PCR. Primers (Tsingke Biotechnology Co., Ltd, Beijing, China) used are displayed in Supplementary Table 2. Clinical characteristics of patient cohort are displayed in Supplementary Table 3.

### Statistical Analysis

All the data were analyzed using the R software version 4.1.0. The OS of the patients with glioma between different groups was analyzed using Kaplan–Meier curves with the log-rank test. Correlations were assessed *via* Spearman's coefficient. Kruskal–Wallis tests were applied for the comparison of gene expression in two or more groups. The landscape of CNV and gene location were visualized by the RCircos R package (31). A *p* < 0.05 was considered as statistically significant. Statistical analyses were performed using GraphPad Prism 9 for rest of the data.

## Results

### Landscape of FRGs in GBM

**Figure 1:**
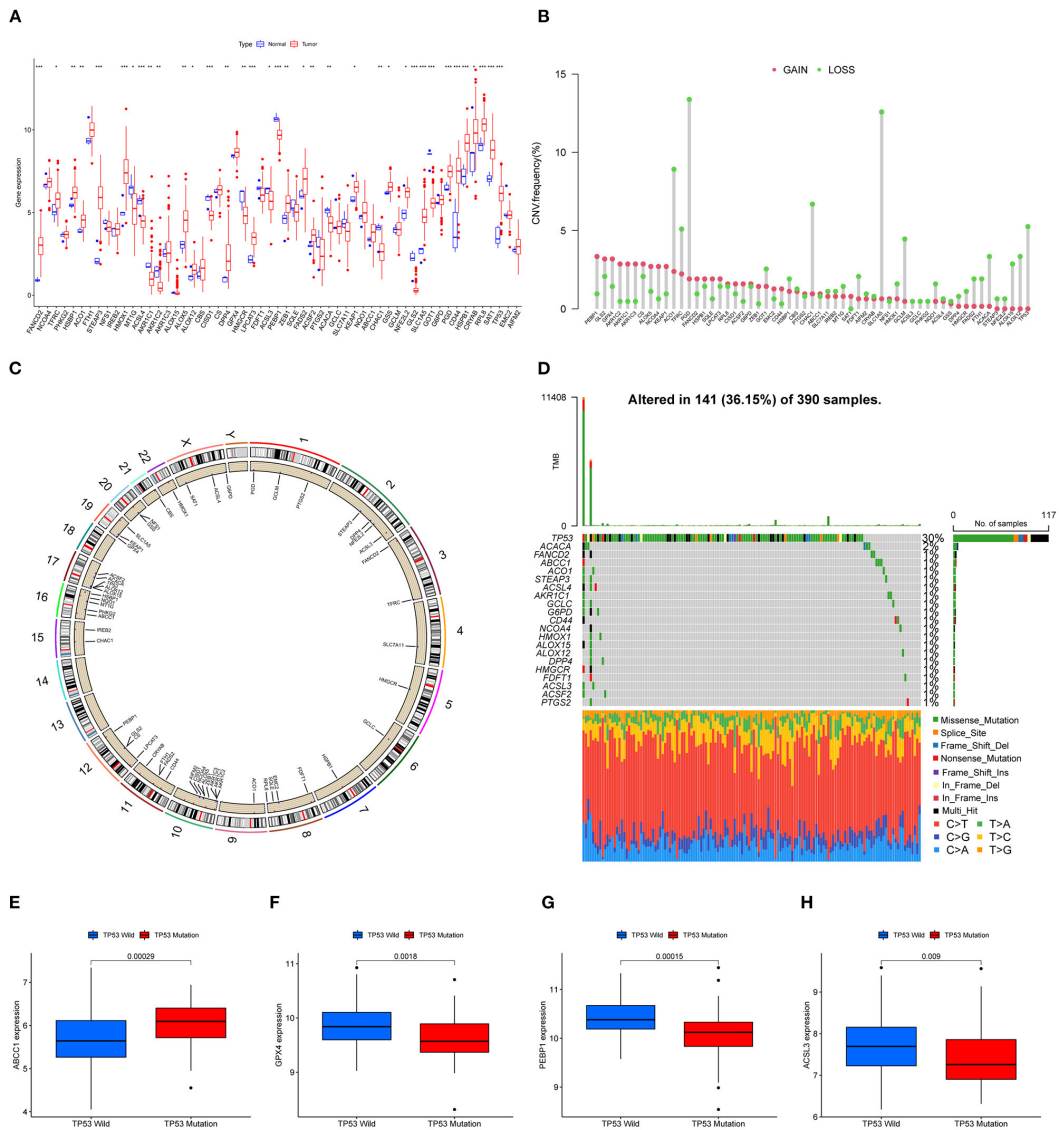
Landscape of FRGs in TCGA-GBM. **(A)** The expression levels of FRGs in the TCGA dataset. Red: upregulated genes; blue: downregulated genes. **(B)** CNV frequency; red: CNV gain; green: CNV loss. **(C)** The CNV distribution of all chromosomes. **(D)** The mutation of FRGs in GBM; green boxes represent missense mutations, orange splicing mutations, blue frameshift mutations, and red nonsense mutations. **(E–H)** Four genes (ABCC1, GPX4, PEBP1, and ACSL3) with significantly different expressions between TP53 wildtype and mutant (*p* < 0.01).

A total of 59 FRGs previously reported were included in this study. We first analyzed the expression of these genes in TCGA-GBM and normal tissues. The expression of 59 genes showed significant differences in TCGA-GBM with normal samples (Figure 1A). Among them, FANCD2, STEAP3, HMOX1, and other eight genes were upregulated in GBM (*p* < 0.001), whereas ACSL4, GLS2, and PEBP1 were the opposite (*p* < 0.001). We next examined CNV and chromosome location. Chromosome 10 carried the largest number of genes that undergo copy number variation. PGD and SLC1A5, the genes with the highest frequency of copy number loss, were located on chromosomes 1 and 19, respectively (Figures 1B,C). After that, we investigated the mutation frequencies of these genes in the TCGA-GBM dataset. As a result, there were 21 FRGs with mutation frequency >1%, and TP53 had the highest mutation frequency which was predominantly missense mutation (Figure 1D). Given the high frequency of copy number loss and mutation of TP53, we further explored the gene expression of FRGs between TP53 wildtype and mutant type. Four genes differentially expressed between subgroups were shown, and ABCC1 exhibited an increased expression in the TP53 mutant group (Figures 1E–H), which may be associated with malignant progression of TP53 mutant status (32).

### The Relationship Between FRGs and Prognosis

**Figure 2:**
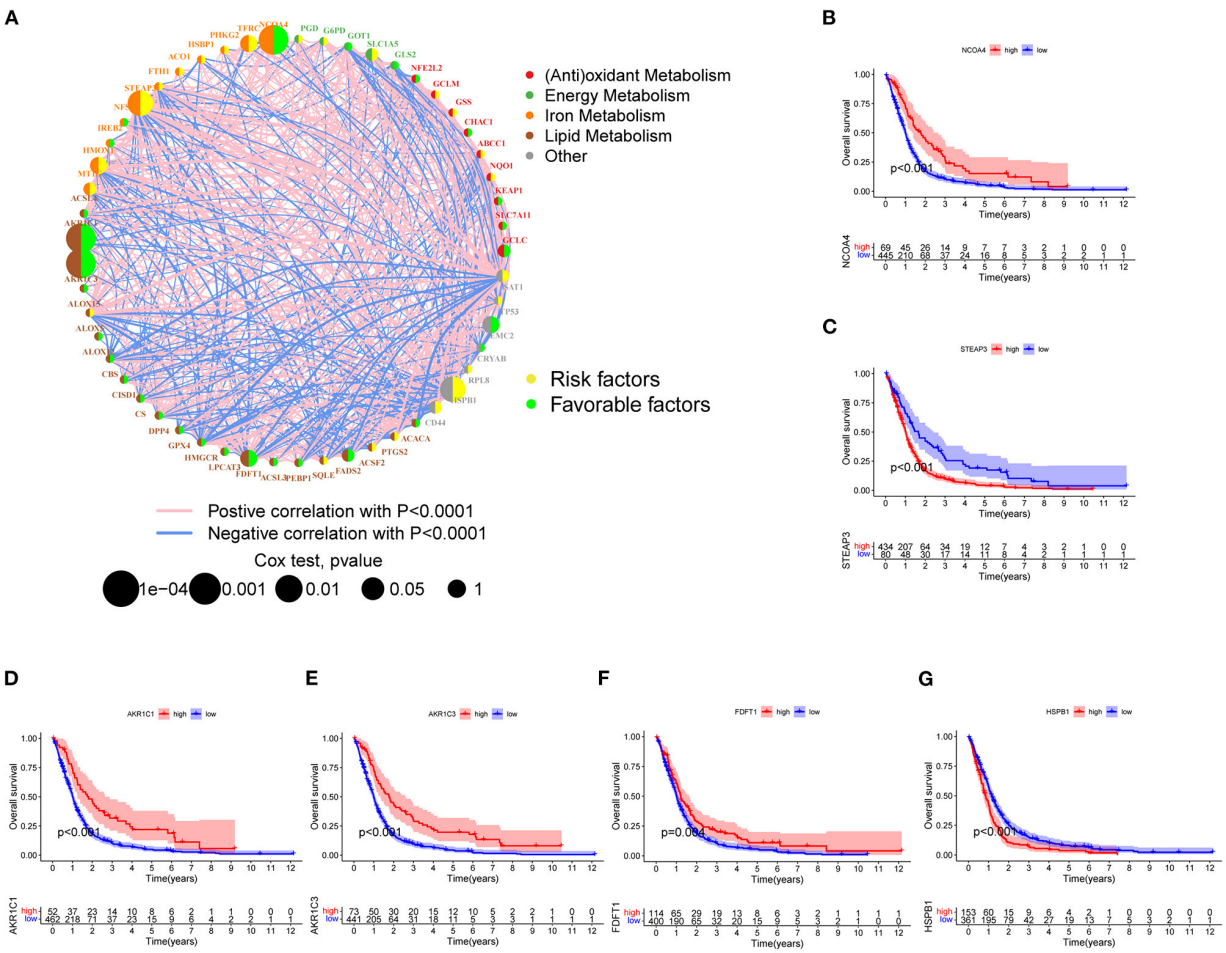
FRGs associated with prognosis. **(A)** The degree distribution of the prognostic-related gene network. The yellow circles indicate high-risk genes, and the green circles indicate low-risk genes. The remaining five colored dots represent five types of FRGs. The blue line indicates a negative correlation, and the pink lines indicate positive correlations with correlation coefficients of *p* < 0.0001. Cox regression analysis (Cox *p*-value range 0.0001–1). The size of the dot reflects the *p*-value. **(B–G)** Six FRGs significantly associated with prognosis (NCOA4, STEAP3, AKR1C1, AKR1C3, and HSPB1 *p* < 0.001; FDFT1 *p* < 0.01).

Three mRNA-seq datasets that include TCGA-GBM (*n* = 161), CGGA325 (*n* = 137), and GSE12041 (*n* = 218) were integrated to interrogate the prognostic significance of FRGs. According to the previous studies of different metabolic pathways, genes related to ferroptosis were preliminarily divided into five categories. Gene expression, correlation, and prognostics are shown in Figure 2A. Among them, AKR1C1, AKR1C3, FDFT1 that involved in lipid metabolism, and NCOA4 that involved in iron metabolism were significantly associated with improved prognosis, which can be regarded as the protective factors. In contrast, the expression of STEAP3, HMOX1 that involved in the iron metabolism, and HSPB1 and SAT1 belong to other categories was associated with poor prognosis, which can be regarded as the risk factors (Figure 2A). Next, the Kaplan–Meier survival curve was used, and six genes that include NCOA4, STEAP3, AKR1C1, AKR1C3, FDFT1, and HSBP1 were most significantly related to OS (Figures 2B–G), which indicates that they may be vital in predicting patient prognosis.

### GSVA and ssGSEA Analysis in three Clusters of FRGs

**Figure 3:**
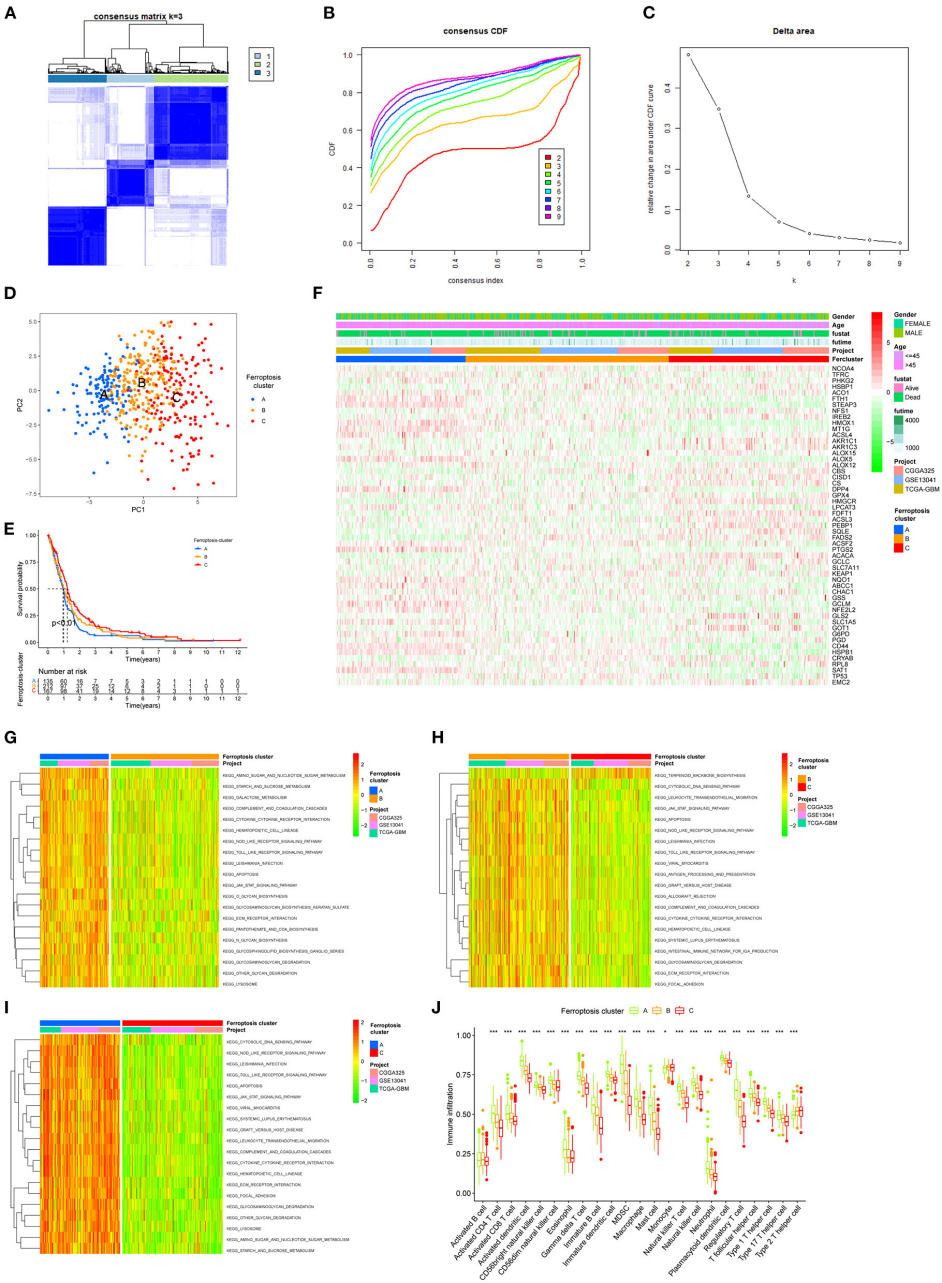
The unsupervised clustering process for FRGs. **(A)** Consensus clustering matrix for k = 3. **(B)** Consensus clustering CDF for k = 2–9. **(C)** Relative change in area under the CDF curve for k = 2–9. **(D)** A PCA plot of unsupervised clustering when k = 3. **(E)** Prognostic differences between the three clusters after merging survival information (*p* < 0.01). **(F)** The heatmap of three clusters and their clinical characteristics. **(G–I)** Visualization for the results of KEGG for DEGs with three clusters. **(J)** The relative enrichment of each immune cell fraction in the TIME with the gene sets using ssGSEA (*p < 0.05, **p < 0.01, ***p < 0.001).

To characterize the functions of these FRGs in GBM, they were clustered for further analysis (Figure 3A). The consensus distributions for *k* (2 to 9) were displayed in the empirical cumulative distribution function (CDF) plots (Figures 3B,C); given the consensus matrix, *k* = 3 seemed to be the most suitable choice. Besides, to verify the effectiveness of unsupervised clustering, principal component analysis (PCA) can clearly show the distinction between 3 clusters which proved the accuracy of our selection for k and the effectiveness (Figure 3D). K–M analysis found significant differences in OS in the three clusters (*p* = 0.008), and cluster A seemed to have the poorest prognosis. Next, the expression of FRGs in the three clusters and their clinical characteristics were shown in the heatmap (Figure 3F). To gain insights into the functional implication, GSVA was performed to analyze the differentially enriched KEGG pathways in two of any three clusters. Samples in cluster A showed prominent enrichment of nod-like receptor (NLR) signaling pathway, apoptosis, amino sugar and nucleotide sugar metabolism, and cytokine–cytokine receptor interaction, etc. (Figures 3G–I). Finally, enrichment of immune cell fractions in the tumor immune microenvironment (TIME) was assessed using the ssGSEA algorithm. As a result, most types of immune cells were significantly enriched in cluster A, such as activated CD8 T cell and eosinophil (Figure 3J). These findings indicated that different regulatory patterns based on FRGs reflected the mechanisms in tumor growth, apoptosis, and immune infiltration.

### Identification of Hub Genes in DEGs Using WGCNA and Functional Annotation

**Figure 4:**
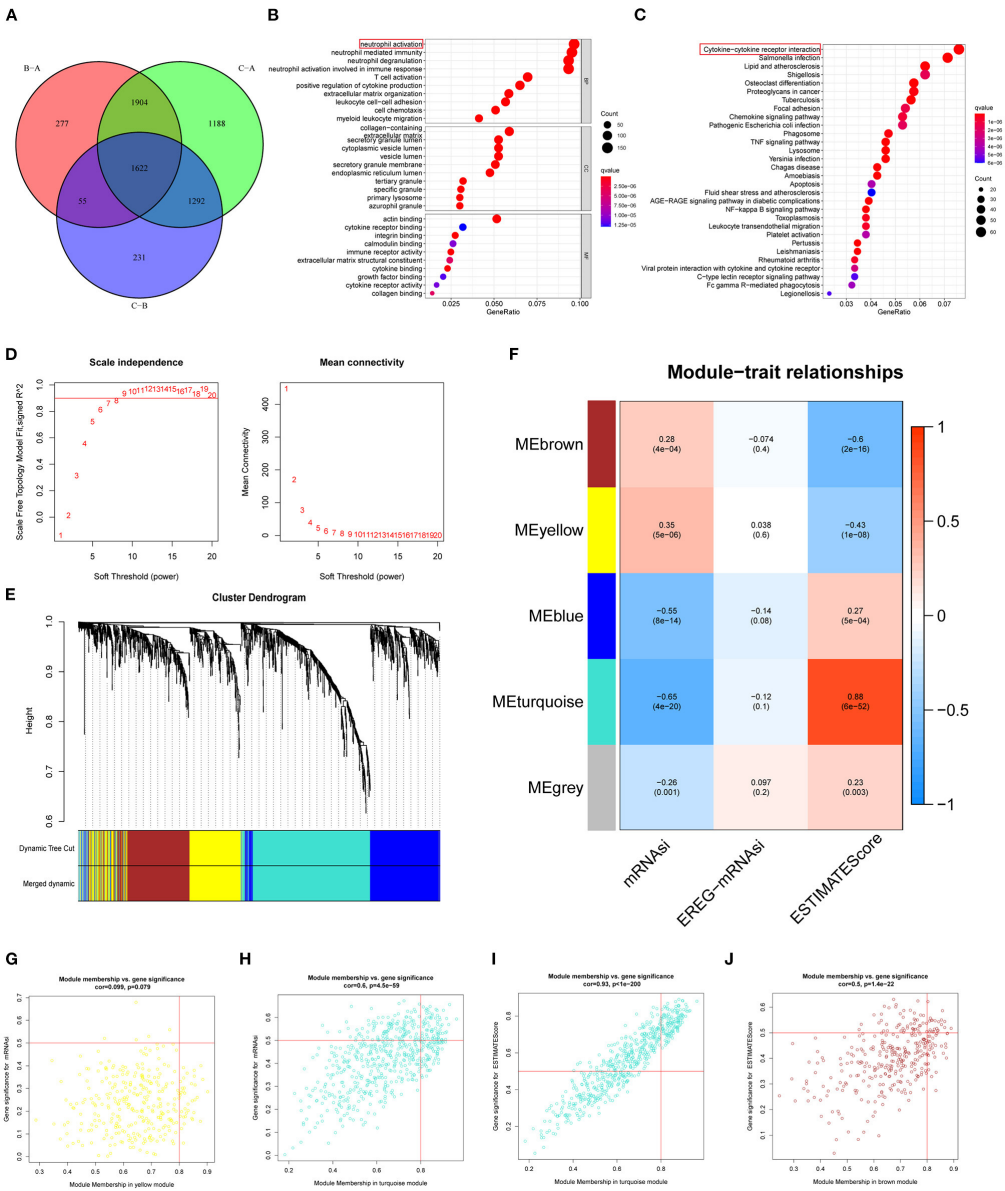
Characteristics of potential traits in different patterns of ferroptosis regulation. **(A)** A Venn diagram of differential genes in three patterns (*p* = 0.001). **(B)** GO analyses of differentially expressed. **(C)** KEGG pathway analyses of differentially expressed. **(D–F)** Hierarchical clustering dendrograms of identified coexpressed genes in modules. The branches of the cluster dendrogram correspond to the different gene modules. Each leaf corresponds to a gene. Each colored row represents a color-coded module, which contains a group of highly connected genes. A total of 5 modules were identified after the merger. **(F)** Correlations between the gene modules and target traits including mRNAsi, EREG-mRNAsi, and ESTIMATEScore. The corresponding *p*-value is increased in size from blue to red. **(G–J)** The four modules most significantly correlated with mRNAsi and ESTIMATEScore. Cor was the coefficient indices and *p* was Pearson's correlation.

To investigate the specific phenotype-related genes for each regulatory pattern of ferroptosis, we used the “LIMMA” package to identify the DEGs. A total of 1,622 DEGs were picked out in three clusters (Figure 4A). Functional enrichment analysis found that the main function of these DEGs enriched in neutrophil activation neutrophil-mediated immunity (BP), collagen-containing extracellular matrix, secretory granule lumen (CC), and actin-binding (MF) (Figures 4B,C). For the KEGG pathway, the most significant pathway for these DEG enrichment was cytokine–cytokine receptor interaction. Next, WGCNA was performed to structure gene coexpression networks and further identified biologically meaningful modules that corresponded to designate phenotype-related genes, which include stemness indices and the ESTIMATEScore. The most appropriate β was 7, and the relatively balanced scale independence and mean connectivity of the WGCNA were identified (Figure 4D). A total of 5 modules (merged dynamic) were identified (Figure 4E minModuleSize = 50). To analyze the correlations between merged modules and the immune and stemness phenotypes, module eigengenes (MEs), which could be regarded as representative of the gene expression patterns in a module, were determined and used to calculate the correlations with designated phenotypes. The heatmap in Figure 4F revealed the key modules (MEyellow and MEturquoise for the mRNAsi and MEturquoise and MEbrown for the ESTIMATE). The correlation graphs were plotted to select the hub genes in these modules (Figures 4G–J). Interestingly, the module turquoise had the most positive correlation with ESTIMATEScore and most negative correlation with mRNAsi. Genes with p.MMturquoise ≥ 0.8 and GS.mRNAsi ≤ −0.5 or GS.ESTIMATEScore ≥ 0.5 were screened out (Supplementary Table 4).

**Figure 5:**
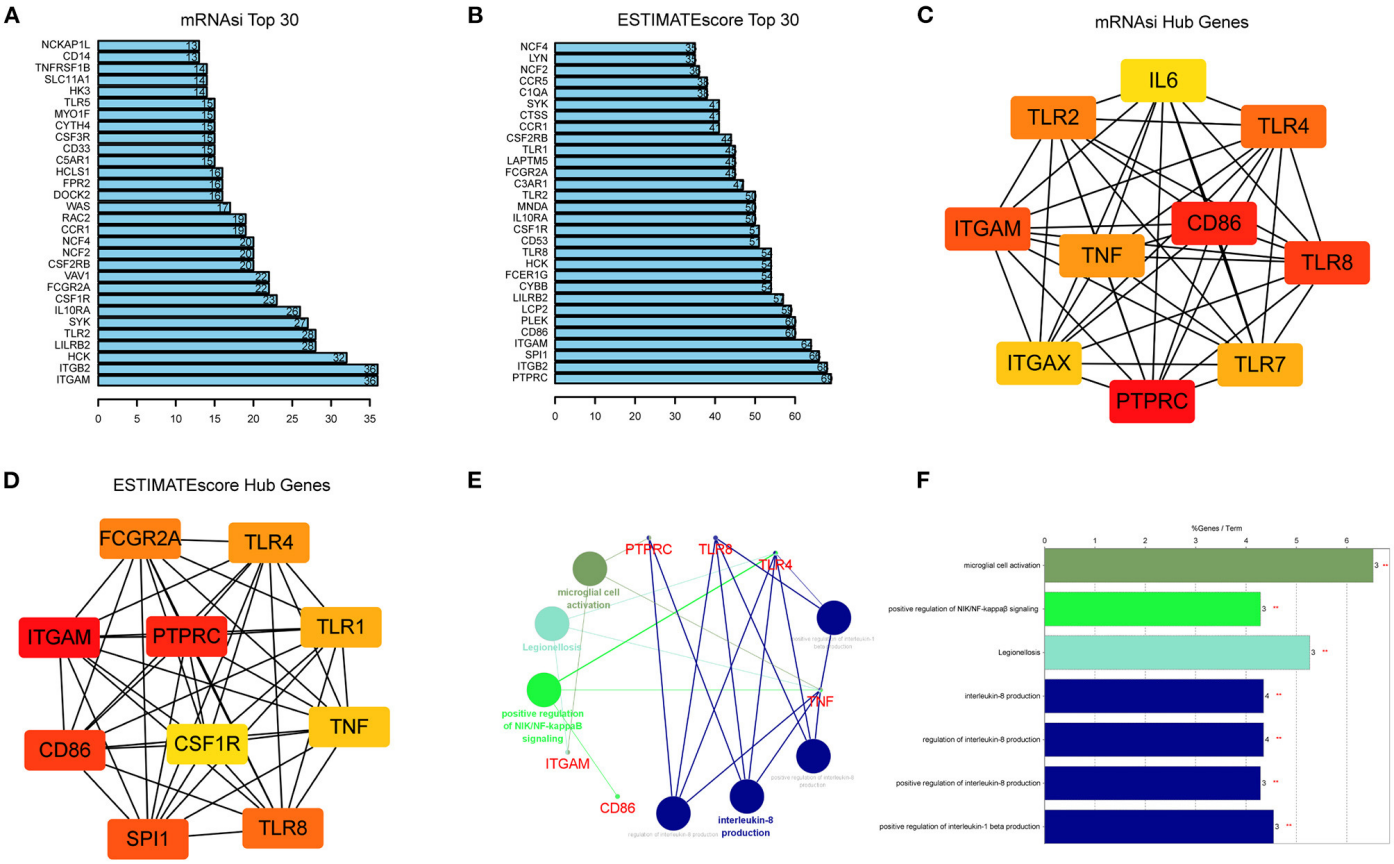
Identification of hub genes from the PPI network. **(A,B)** PPI network obtained using STRING and showing the top 30 highest scoring genes. The bar plots of top 30 key genes of mRNAsi **(A)** and ESTIMATEScore **(B)**. **(C,D)** Top 10 hub genes of mRNAsi **(C)** and ESTIMATEScore **(D)** explored by CytoHubba algorithm in Cytoscape software 3.8.0. and red nodes represent hub genes. **(E,F)** KEGG pathway and GO analysis of five overlapping genes (TLR4, TLR8, TNF, CD86, ITGAM, and PTPRC) using the “ClueGO” and “CluePedia” plugins (*p* < 0.05).

The STRING was used to construct PPI networks. In the module turquoise, 71 genes are negatively correlated with mRNAsi, and 134 genes are positively correlated with ESTIMATEScore (GS > 0.5 and MMturquoise > 0.8). According to the confidence, the genes with significant interaction were screened, and the filtered results were imported into the Cytoscape for network visualization. The top 30 of these genes associated with mRNAsi and ESTIMATEScore with the highest combined score in STRING are exhibited (Figures 5A,B). CytoHubba and Maximal Clique Centrality (MCC) were used to explore important nodes. Top 10 MCC values were selected, and then, the intersection was taken to get the hub genes in PPI analysis (Figures 5C,D). As the result, we found that 5 genes, which include TLR4, TLR8, TNF, CD86, ITGAM, and PTPRC, were the common hub genes in the two modules. These five key genes were analyzed by KEGG and GO enrichment using the “ClueGO” and “CluePedia” plugins for Cytoscape software (Figures 5E,F *p* < 0.05). We found that these genes showed the enrichment of GO terms related to microglial cell activation, positive regulation of NIK/NF-kappaβ signaling and interleukin (interleukin-8 production and regulation and positive regulation of interleukin-1 beta production). Collectively, these hub genes may be the key components of the GBM immune and stemness module that contribute to immunoregulatory functions during ferroptosis.

### Cluster Analysis of DEG Levels

**Figure 6:**
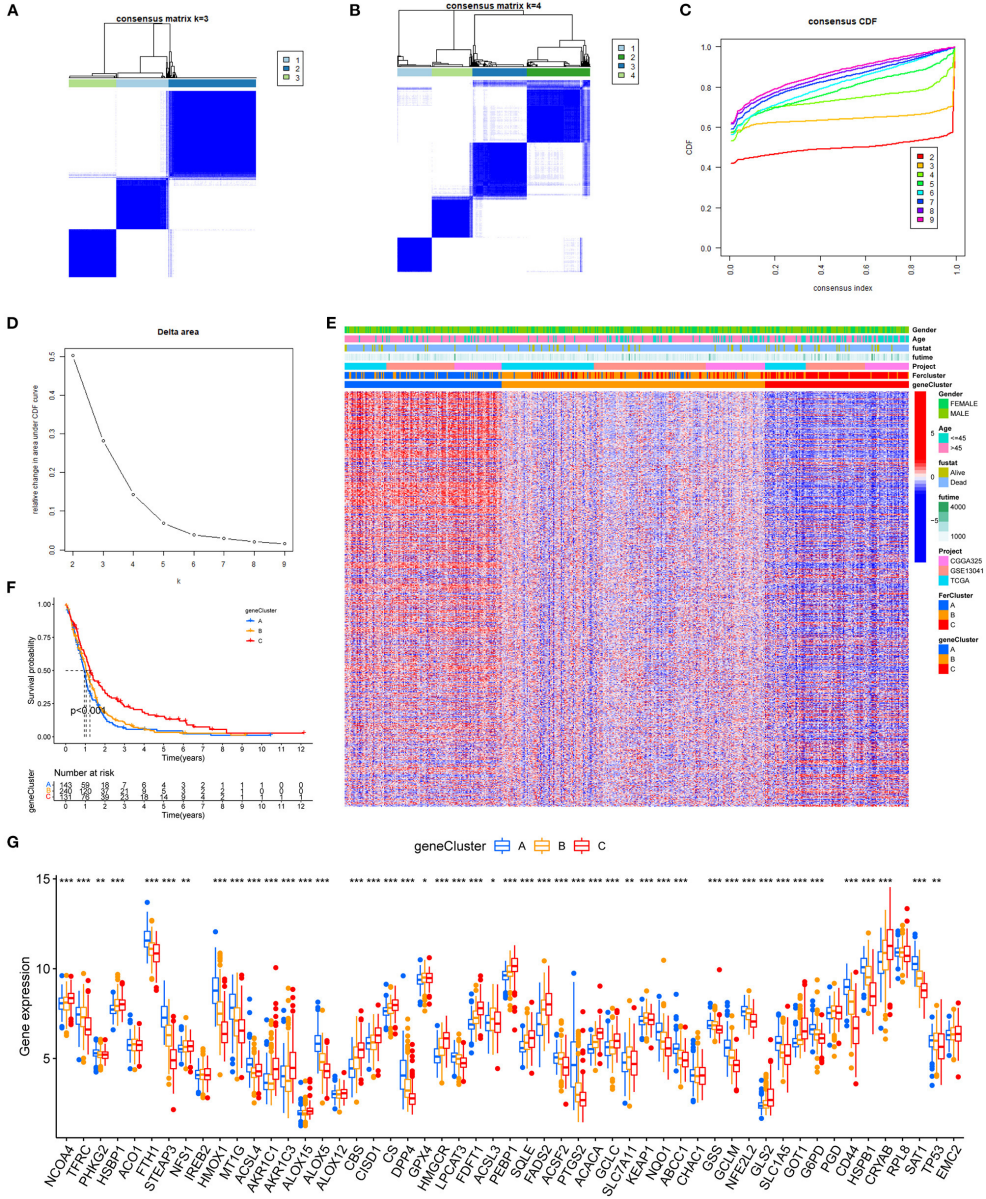
Hierarchical cluster analysis of DEGs. **(A–D)** The consensus clustering matrix for *k* = 3 was determined by CDF for *k* = 2–9. **(E)** Prognostic differences between the three clusters after merging survival information (*p* < 0.01). **(F)** The heatmap of three clusters and their clinical characteristics. **(G)** The expression levels of FRGs in different clusters (*p < 0.05, **p < 0.01, ***p < 0.001).

Previous results revealed the special performance of the DEGs in immunity and stemness of GBM. To further study, the association between these DEGs and FRGs was clustered into three categories according to their correlations in GBM. Given the consensus matrix for the analysis, *k* = 3 seemed to be the most suitable choice (Figures 6A–D). Similarly, these clusters also showed significant survival differences (Figure 6F *p* < 0.001). The differences in survival obtained by this clustering are consistent with those using FRGs (Figure 3E). The expression of DEGs in the clustering of the two methods and their clinical characteristics were shown in the heatmap (Figure 6E), and these findings were independent of clinical traits, such as age and sex. For FRGs, we found that most genes show significant differences in DEG clusters (Figure 6G). Interestingly, among the FRGs, FTH1, STEAP3, HMOX1, and 13 other genes showed an increased expression in cluster A, which had the worst prognosis.

### Construct the FRG Prognostic Signature

**Figure 7:**
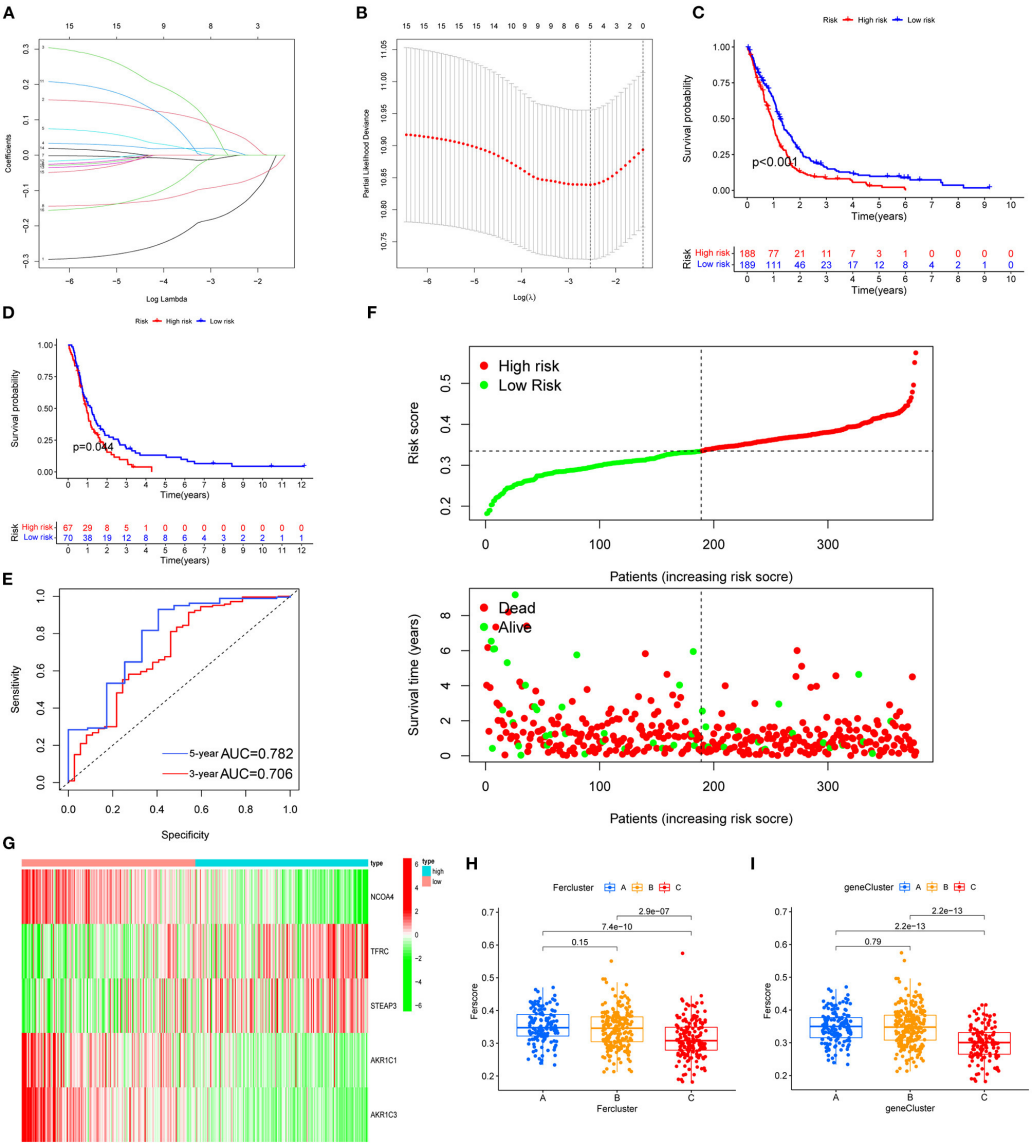
FRG prognostic signature. **(A,B)** The process of building the signature. LASSO regression was performed, calculating the minimum criteria. **(C,D)** Kaplan–Meier curves showed that the high-risk subgroup had worse OS than the subgroup in training set (**C**, *p* < 0.001) and test set (**D**, *p* < 0.001). **(E)** ROC curves showed the predictive efficiency of the risk signature on the 3-year and 5-year survival rates of training set (3-year AUC = 0.706, 5-year AUC = 0.782). **(F)** The distributions of risk scores and the distributions of risk scores and OS status. The green and red dots indicated the alive and dead status, respectively. **(G)** The heatmap based on the expression of the five genes in the high- and low-risk group. **(H,I)** According to the formula, the different clustered samples are scored using the coefficients.

**Figure 8:**
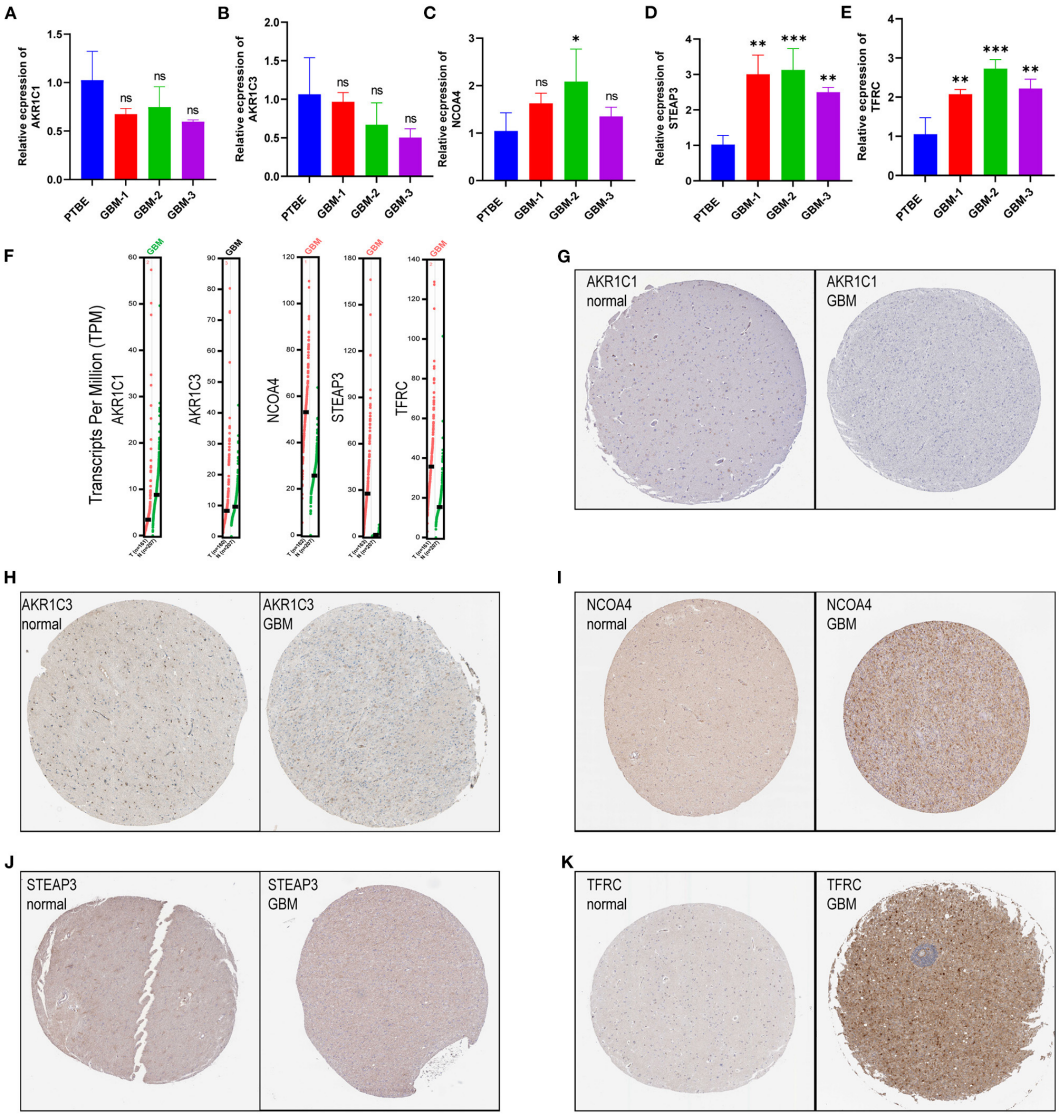
Validation of risk genes **(A–E). (A)** AKR1C1, **(B)** AKR1C3, **(C)** NCOA4, **(D)** STEAP3, and **(E)** TFRC expression in peritumoral brain edema and GBM tissues. **(F)** The expression of prognostic model with five genes (NCOA4, TFRC, STEAP3, AKR1C1, and AKR1C3) in GEPIA. **(G–K)** Validation of five genes with immunohistochemistry from the HPA database.

Among the genes related to prognosis (Figure 2A), 16 FRGs were correlated with the OS of GBM patients. LASSO Cox analysis was performed to establish an FRG prognostic signature. In the crossvalidation process, lambda.Min was regarded as the optimal value (Figures 7A,B). A number of 5 FRGs were identified, and corresponding coefficients were calculated. A number of 516 samples were divided into train set (TCGA-GBM and GSE13041) and test set (CGGA-325), and samples were split into high- and low-risk subgroups by the median value of the risk score. Kaplan–Meier survival curves depicted that GBM patients with increased risk scores had worse clinical outcomes (Figures 7C,D, *p* < 0.001 in both train and test datasets). Next, we established 3- and 5-year ROC curves and found that the risk score can effectively distinguish GBM patients with different survival statuses in train set (Figure 7E, 3-year AUC = 0.706, 5-year AUC = 0.782). The risk score and survival status distributions of the train set are shown in Figure 7F. The mortality of patients increased with the increase of the risk score. The expressions of risk genes and protective genes in these 5 genes are shown in the heatmap (Figure 7G), TFRC, and STEAP3 as the risk factors increased in the high-risk score group. Conversely, NCOA4, AKR1C1, and AKR1C3 become the protective factors. The risk scores in the ferroptosis cluster and gene cluster are shown in Figures 7H,I. Univariate and multivariate Cox regression analyses show the independent prognostic value of this risk score (Supplementary Figures 1A,B). A Nomogram model was established which contained risk score, recurrent, age, and gender to assess the survival prediction in GBM patients (Supplementary Figure 1C). A Sankey diagram is used to link clustering, scoring, and survival status (Supplementary Figure 2). Most of the surviving patients belong to the low-risk group and cluster C. Finally, considering the small number of normal samples in the dataset, we validated the expression of the five key genes by qRT-PCR with the unpaired *t*-test in human GBM tissues. The results of qRT-PCR (Figures 8A–E) and dates in GEPIA (Figure 8F) were consistent with the expression of protein in HPA (Figures 8G–K).

### Enrichment Analyses of Immune-Related Functions

**Figure 9:**
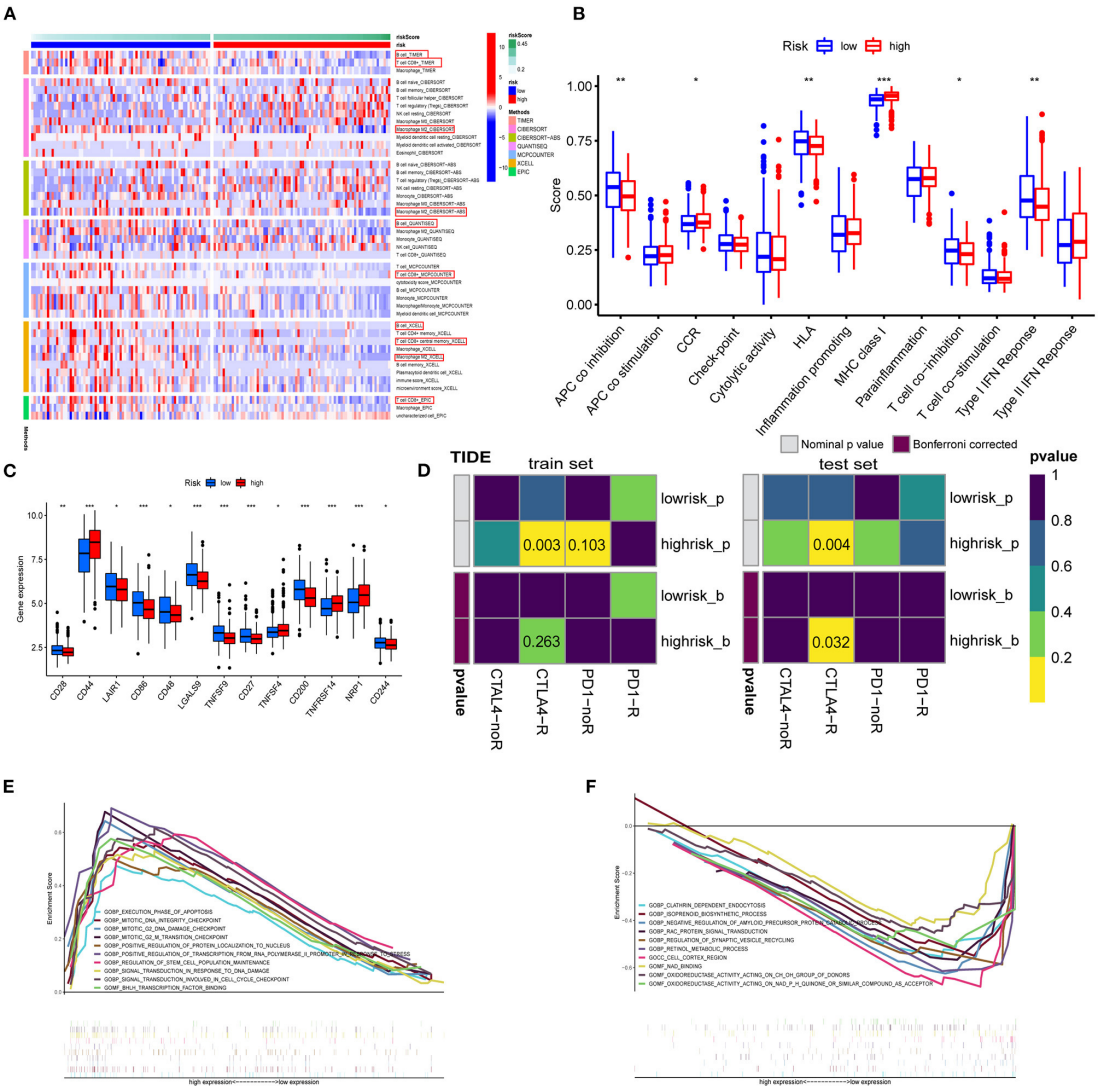
Enrichment analysis of immune-related functions. **(A)** The TIMER, CIBERSORT, CIBERSORT.ABS, QUANTISEQ, MCPCOUNTER, XCELL, and EPIC algorithms were applied for the immune infiltration of the high- and low-risk groups. **(B)** Immune score estimated by single sample GSEA (ssGSEA) in different groups. **(C)** Immune checkpoints in the tumor microenvironment. **(D)** TIDE prediction of the association of genes prognostic signature with ICB responsiveness in train and test sets. **(E,F)** GO analysis for high-risk group **(E)** and low-risk group **(F)** using GSEA 4.1.0.

The enrichment scores of immune cells and corresponding immune functions and pathways with ssGSEA were quantified for the TCGA dataset. Silico approaches that include TIMER, CIBERSORT, CIBERSORT–ABS, QUANTISEQ, MCPCOUNTER, XCELL, and EPIC computational were employed to quantify the immune cells in high- and low-risk groups (Figure 9A). Consequently, the fraction of B cell, CD8^+^ T cell, and M2 macrophage were significantly increased in the low-risk group. NK cell and T cell regulatory (Tregs) were enriched in the high-risk group. The MHC class I scored higher in the high-risk group. The GSVA method was used to calculate the immune event scores of the high- and low-risk groups. APC coinhibition, HLA, and type I IFN response scored higher in the low-risk group (Figure 9B, *p* < 0.01). Next, we explored the relationship between immune checkpoint-related genes and risk score (Figure 9C). The expression of CD44, TNFRSF14, and NRP1 in the high-risk group was significantly higher than that in the low-risk group. Given this, we introduced the TIDE algorithm to assess the efficacy of FRG signatures in predicting ICB responsiveness in GBM. Submap was used to compare the prediction results (Figure 9D). As a result, different groups in train and test sets showed comparable performance in predicting the GBM response to anti-CTLA4 therapy (*p* < 0.05). Finally, we use GSEA to perform GO enrichment analysis on high- and low-risk GBM patients. The samples of the high-risk group were enriched in positive regulation of transcription from RNA polymerase II promoter in response to stress (GOBP), mitotic G2 M transition checkpoint (GOBP), BHLH transcription factor binding (GOMF) (Figure 9E). In the low-risk group, enriched GO terms were cell cortex region (GOCC), negative regulation of amyloid precursor protein catabolic process (GOBP), and oxidoreductase activity acting (GOMF). In conclusion, GBM patients with high- and low-risk scores had different immune-related functions (Figure 9F).

## Discussion

Malignant glioma remains a considerable threat to human health, and the prognosis of patients with GBM is dismal (33, 34). Recently, regulated cell death has gained considerable attention in cancer, especially ferroptosis (7, 35, 36). Herein, 59 FRGs in GBM were included in this study to investigate the characteristics with expression, OS, and functions. CNV exists as a genetic polymorphism in the human genome, and CNV alters tumorigenesis by deletion or amplification of a copy number of a gene (37, 38). In this study, the highest frequency of FRG PGD and SLC1A5 CNV (loss) was located on chromosomes 1 and 19, respectively. SLC1A5 expression correlated positively with immune cells, such as tumor-infiltrating B cells, CD4^+^ T in hepatocellular carcinoma, and lower-grade glioma (39). For mutations in FRGs, TP53 mutation is one of the most frequent genetic alterations in primary glioma. Previous studies have shown that TP53 polymorphism is associated with the risk of primary glioma (40). Of note, the expression of ABCC1 was higher in mutant TP53 whereas the expression of GPX4, PEBP1, and ACSL3 were higher in the wild-type TP53 group. The finding implicated TP53 mutation status was an important link in the regulation of other FRGs.

Unsupervised cluster analysis of the expression values of FRGs identified three distinct patterns in GBM. Cluster A with the worst prognosis showed high enrichment in NLR signaling pathway apoptosis, and amino sugar and nucleotide sugar metabolism. The NLR family of receptors had been recognized as the key roles of immunity and inflammation with GBM (41). Meanwhile, a variety of immune cells, such as activated CD8 T cell and eosinophil, exhibit aggregation in cluster A. Whether a direct mechanism of immune cells on ferroptosis nodes might be of physiological relevance remains elusive. Recent studies have shown that CD8 T cells may in sensitizing tumor cells toward ferroptosis (42). In addition, 1,622 DEGs were selected from three patterns. GO and KEGG pathway analysis revealed that DEG enrichment was mainly involved in the immunity biological process. Activated neutrophils are induced by the microenvironment of GBM (43). Meanwhile, some immune-associated lncRNAs in glioma were verified to be closely related to cytokine–cytokine receptor interaction (44). Functional annotation of the hub genes identified by WGCNA illuminated the potential regulatory mechanisms by which of FRGs regulate on the immune and stemness phenotypes. Recent studies suggest a possible negative regulation between stemness and immune activation (45). Glioblastoma multiforme stem cells, characterized by self-renewal and therapeutic resistance, play vital roles in GBM (6). Using the app CytoHubba in Cytoscape, we filtered 5 hub genes in both immune and stemness PPI networks. TLRs are expressed on both immune and tumor cells, which play dual roles in countering cell proliferation, migration, invasion, and glioma stem cell maintenance responses (46). TNF-α/NF-κB signaling is closely associated with glioma proliferation (47). Also, CD86 is an unfavorable prognostic biomarker in lower-grade glioma (48). It is worth noting that PTPRC, TLR8, TLR4, and TNF all exhibit functions related to IL-8 regulation. Interleukin-8 (IL-8) has been revealed as a critical regulator of central nervous system (CNS) function and development with participation in many CNS disorders including gliomas (49, 50). These are the key genes of immunity and stemness from different ferroptosis regulation patterns. This suggests that among the multiple FRGs, some genes regulate GBM stem cells and the immune microenvironment. The connection between them is also the direction of our next research.

Based on five FRGs, a prognostic model was established and validated in TCGA-GBM, GSE13041, and CGGA-325. These databases have authoritative gene expression and clinical information for GBM. The prognostic model contained five genes (NCOA4, TFRC, STEAP3, AKR1C1, and AKR1C3). The mRNA expression of genes was verified using qRT-PCR. Based on the HPA database, genes were verified at the protein levels. These FRGs affect many of the key processes involved in the tumorigenesis and progression of cancer, especially glioma. NCOA4 is a selective cargo receptor for the autophagic degradation of ferritin in glioma which is known as ferritinophagy (16, 51). The TFRC expression was higher in glioma (52), and the progression and oncogenicity of glioma were regulated by hsa-miR-144-3p/TFRC signaling (53). STEAP3 emerged as an important protein that induces mesenchymal transition and stem-like traits in glioma (54). AKR1C1 and AKR1C3 are members of the AKR superfamily which has been previously shown to be associated with oncogenic potential and proliferation capacity (55), and selective targeting of AKR1C proteins in GBM could delay the acquisition of resistance to TMZ of astroglioma cells (56). This prognostic model could predict tumor prognosis, and targeting these prognostic model genes may provide new ideas for the development of targeted treatment tools.

Our results demonstrated that a high-risk score was associated with a worse prognosis. Three-year and 5-year ROC curves indicated the 5-gene signature as a potential diagnostic factor in GBM patients. Moreover, outcome of the nomogram showed that risk score and age were associated with GBM prognosis, and it was consistent with the actual clinical situation. We investigated the correlation between high- and low-risk groups and immune cells with the CIBERSORT, CIBERSORT-ABS, QUANTISEQ, XCELL, MCPCOUNTER, and EPIC algorithms. Tregs were elevated in the high-risk group. The findings of this study are in line with those presented in previous studies, Tregs play important known roles in suppressing the immune response and maintaining immune homeostasis (57). Innovatively supporting that the abundance of nonpolarized M0 macrophages rather than M1 or M2 macrophages assembly in glioblastoma that contributed to the malignancy of tumor was proposed recently (58). Also, a recent mice study showed that increasing glioma-associated monocytes in intracranial murine GL261 leads to an increase in intratumoral and systemic myeloid-derived suppressor cells (59). In summary, regulation of ferroptosis in GBM patients may be important in controlling the inflammatory and immune responses. Research on immune checkpoints has now become a new hotspot. In this study, significant differences in the expression of immune checkpoints between high- and low-risk groups suggested that the sensitivity to immunotherapies is associated with a risk score. Moreover, our risk score may screen out potential ICB responders. This provides a new idea for *in-vivo* experiments of immunotherapy. However, GBM patients with low OS exhibit higher expression of markers characterizing immune response activity and T cell infiltration (60). Besides, the presence of the blood–brain barrier cannot be ignored for the nature of immunotherapy. In fact, considering that targeting these FRGs indirectly improves immunotherapy, many questions need answering.

This study still has some limitations. First, all the data used to construct and validate the prognostic model were obtained from publicly available datasets. These three GBM databases inevitably lead to the neglect of intra-tumor heterogeneity in different databases. As confirmed in the study, tumor heterogeneity has an important impact on diagnosis and treatment (61). A prospective study is needed to assess the potential application of the signature. Second, for the five key genes related to GBM stemness, which FRGs or pathways regulate them remains to be further elucidated. Third, although the survival benefits and immune-related biological processes with ferroptosis-related gene signature have been revealed through functional analysis, *in-vivo* and *in-vitro* experiments are needed to further elucidate the specific mechanism, preferably at the single-cell level in humans. Finally, we expect that this work will provide clues on immunity, stemness, and prognosis characteristics for future studies.

## Conclusions

In summary, by analyzing the expression of ferroptosis-related genes in GBM, we identified three ferroptosis regulation patterns of GBM patients. Comparison of the DEG of three patterns and unveiled five key genes involved in immunity and stemness. A prognostic model based on five FRGs was built. The risk score can be a good predictor of prognosis and also predicts the degree of immune infiltration and ICB responsiveness.

## Data Availability Statement

Publicly available datasets were analyzed in this study. This data can be found at: The Cancer Genome Atlas (TCGA) database, https://portal.gdc.cancer.gov/, TCGA-GBM, Chinese Glioma Genome Atlas (CGGA), http://www.cgga.org.cn/, CGGA-325, and National Center for Biotechnology Information (NCBI) Gene Expression Omnibus (GEO), https://www.ncbi.nlm.nih.gov/geo, GSE13041.

## Ethics Statement

The studies involving human participants were reviewed and approved by Institutional Ethics Committee of the Second Affiliated Hospital of Harbin Medical University. The patients/participants provided their written informed consent to participate in this study.

## Author Contributions

SH, JD, and HZ conceived and designed the study and revised the manuscript. JD, FW, JJ, HJ, and XY provided analytical technical support. JD, NW, and JZ participated in the production of charts and pictures. SH supervised the study. JD designed and completed qRT-PCR experiments. All authors have read and approved the final manuscript.

## Funding

This work was funded by the National Natural Science Foundation of China (no. 61575058).

## Conflict of Interest

The authors declare that the research was conducted in the absence of any commercial or financial relationships that could be construed as a potential conflict of interest.

## Publisher's Note

All claims expressed in this article are solely those of the authors and do not necessarily represent those of their affiliated organizations, or those of the publisher, the editors and the reviewers. Any product that may be evaluated in this article, or claim that may be made by its manufacturer, is not guaranteed or endorsed by the publisher.

## Supplementary Material

Supplementary Figure 1 Independent prognostic value of the risk score in the train set and construction of the predictive nomogram. **(A)** Univariate Cox regression analysis of the signature. **(B)** multivariate Cox regression analysis. **(C)** A nomogram of the risk score for predicting 1-, 3-, and 5-year survival.

Supplementary Figure 2 Sankey diagram shows the results of our overall study. Different ferroptosis regulation patterns, three clusters of DEGs, risk scores, and survival status were linked together.

Supplementary Table 1 FRGs and classification.

Supplementary Table 2 Primer sequences for qRT-PCR.

Supplementary Table 3 Clinical characteristics of patient cohort.

Supplementary Table 4 Eligible genes in the modules.
