# Supplementary figures and images for: Ferroptosis-Related Gene Contributes to Immunity, Stemness and Predicts Prognosis in Glioblastoma Multiforme
Source: Front Neurol. 2022 Mar 10;13:829926. doi: 10.3389/fneur.2022.829926 (PMC8960280; doi:10.3389/fneur.2022.829926)

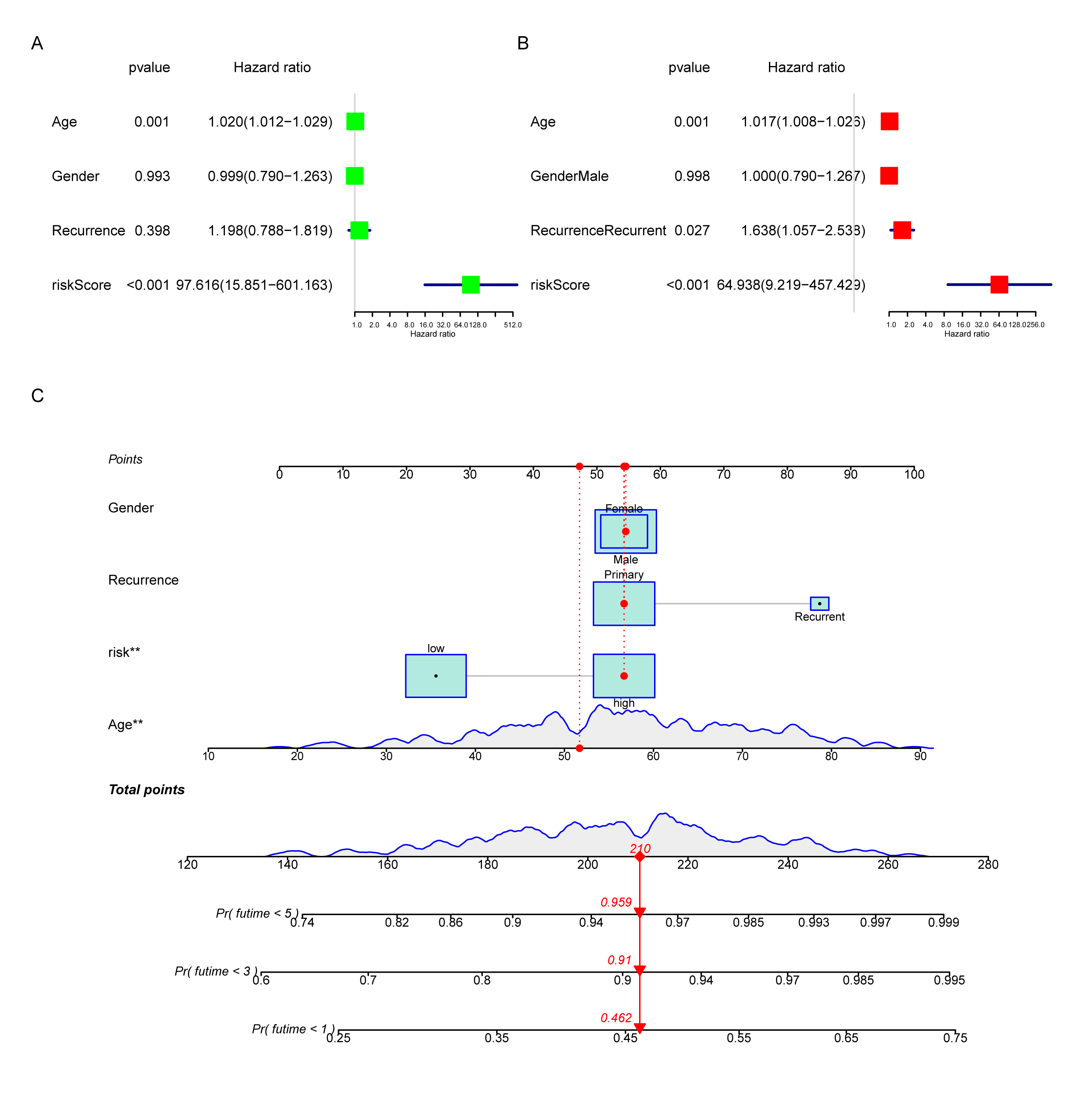

Supplement: Supplementary Figure 1 — Independent prognostic value of the risk score in the train set and construction of the predictive nomogram. (A) Univariate Cox regression analysis of the signature. (B) multivariate Cox regression analysis. (C) A nomogram of the risk score for predicting 1-, 3-, and 5-year survival. [file Image_1.TIF]

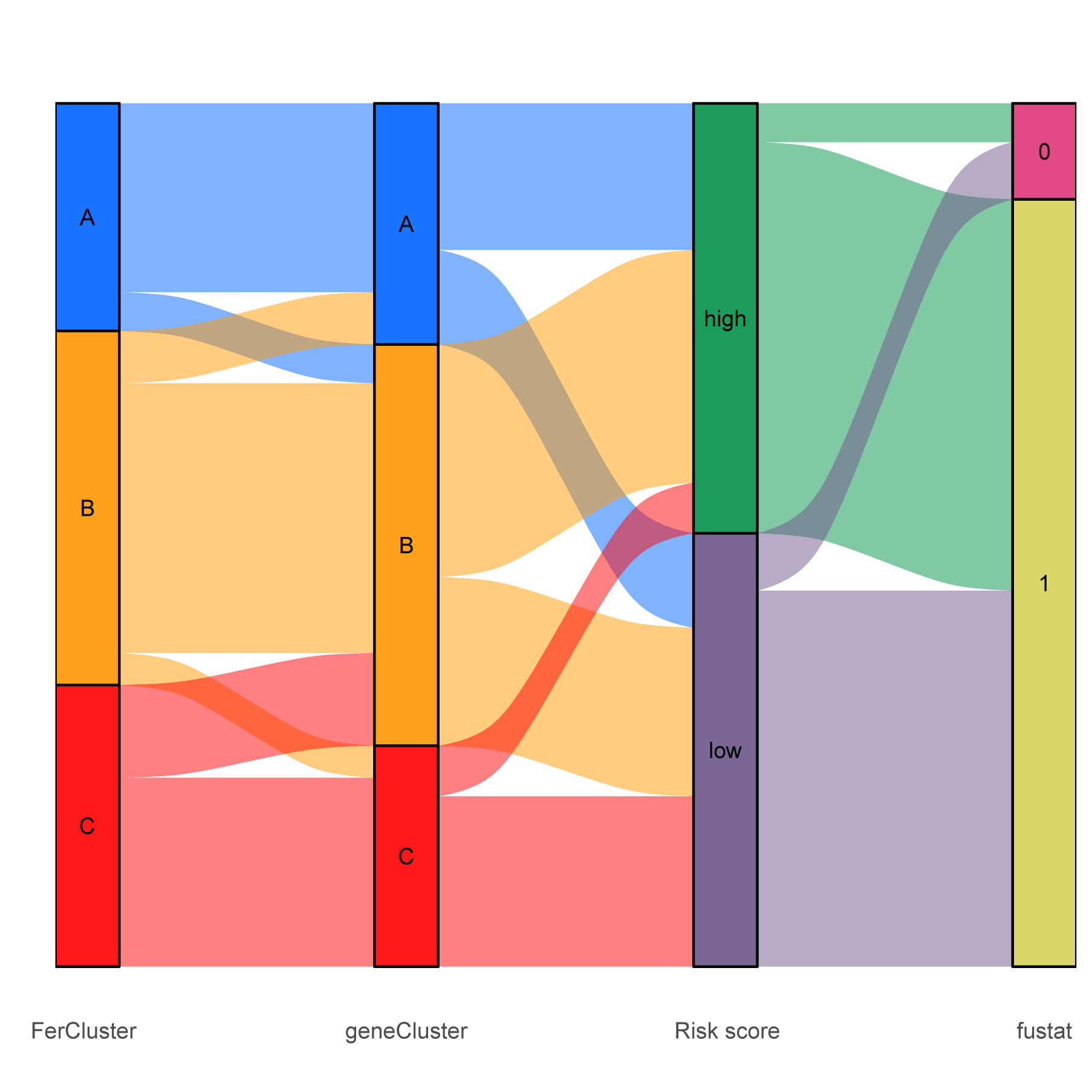

Supplement: Supplementary Figure 2 — Sankey diagram shows the results of our overall study. Different ferroptosis regulation patterns, three clusters of DEGs, risk scores, and survival status were linked together. [file Image_2.TIF]
