# Supplementary material for: Ferroptosis-Related Gene Contributes to Immunity, Stemness and Predicts Prognosis in Glioblastoma Multiforme
Source: Front Neurol. 2022 Mar 10;13:829926. doi: 10.3389/fneur.2022.829926 (PMC8960280; doi:10.3389/fneur.2022.829926)
Supplement: Supplementary Table 2 — Primer sequences for qRT-PCR. [file Table_2.DOCX]

**Table S2. Primer sequences for qRT-PCR.**

| **Targets** | **Forward 5′-3′** | **Reverse 5′-3′** |
| --- | --- | --- |
| AKR1C1 | GGCTTTGTTAGGCAACTGTGTC | ACTGTTGGAAAGGCAGCGAAG |
| AKR1C3 | CCAGTTGACTGCAGAGGACA | GAAGTCGCTAAACAGGACGG |
| NCOA4 | AGAGCAGAAGTCAGCATCCG | AAGCCCTTTAGGTTTCCCCC |
| STEAP3 | GCCCAAGGGATGGTAACAGG | TAGAAGCAGACGAAGAGCCC |
| TFRC | TCCCACCAATCTACACCCCA | CCAATACCGCTGCCTTCTCT |
| β-actin | CCTTCCTGGGCATGGAGTC | TGATCTTCATTGTGCTGGGTG |
